# Continuous surveillance of a pregnancy clinical guideline: an early experience

**DOI:** 10.1186/s13643-017-0506-7

**Published:** 2017-07-14

**Authors:** Laura Martínez García, Hector Pardo-Hernández, Andrea Juliana Sanabria, Pablo Alonso-Coello, on behalf of the Pregnancy Clinical Guideline Updating Working Group, Laura Martínez García, Hector Pardo-Hernández, Andrea Juliana Sanabria, Pablo Alonso-Coello, Longinos Aceituno-Velasco, Ignacio Araya, Emilia Bailon Muñoz, Petra Díaz del Campo, Itziar Etxeandia-Ikobaltzeta, Manuel Fillol Crespo, Lucía Fuertes Luis, Elvira García Álvarez, Laura García Carrascosa, Natalia Gómez-Gómez, Blanca Herrera Crerizo, Jacinta Landa Goñi, Dario López, Arturo Louro González, María Dolores Martínez-Romero, Juliana Ester Martín-López, Adoración Montejo Ráez, Dimelza Osorio, Isabel Roman Romera, Flavia Salcedo-Fernandez, Anna Selva, Ivan Solà, Helen Strivens, Rafael Torrejón-Cardoso, Mar Trujillo-Martín, Robin W. M. Vernooij

**Affiliations:** 1 Iberoamerican Cochrane Centre, Biomedical Research Institute Sant Pau (IIB Sant Pau), Sant Antoni Maria Claret 167, 08025 Barcelona, Spain; 2 CIBER Epidemiología y Salud Pública (CIBERESP), Barcelona, Spain

**Keywords:** Diffusion of innovation, Dissemination and implementation, Evidence-based medicine, Methodology, Practice guidelines, Updating

## Abstract

**Background:**

To date there is no consensus about the optimal strategy for keeping clinical guidelines (CGs) up-to-date. The aims of this study were (1) to develop a continuous surveillance and updating strategy for CGs and (2) to test the strategy in a specific CG.

**Methods:**

The main steps were as follows: (1) recruiting members for the CG Updating Working Group, (2) mapping the CG, (3) identifying new evidence from the CG Updating Working Group, (4) designing and running restricted literature searches, (5) reviewing drugs and medical devices alerts, (6) screening and assessing the new evidence, (7) reviewing and, if necessary, modifying clinical questions and recommendations, and (8) updating the CG document.

**Results:**

The Pregnancy CG Updating Working Group consisted of 29 members, including clinicians, patients and caregivers, and clinical guideline methodology experts. We selected 69 clinical questions (123 recommendations) from the “Assistance during pregnancy” section.

For the first update cycle (32-month duration), 9710 references were identified. Of these, 318 were pertinent, 289 were relevant, and 55 were classified as potential key references. For the second and third update cycles (6-month duration each), 2160 and 2010 references were retrieved, respectively. The continuous surveillance and updating strategy has not yet been completely implemented.

**Conclusions:**

Further resources are needed in updating the CG field, both for implementing updating strategies and for developing methodological research.

## Background

Clinical guidelines (CGs) are useful tools to help patients, health care providers, and policymakers make evidence-based decisions about health care. Consequently, they need to be updated in order to guarantee the validity of their recommendations.

### Time of validity

Several studies have assessed the time of validity of CGs and their recommendations (defined as “time between the publication of a CG and the identification of new relevant evidence”) [1–5]. Data showed that recommendations quickly became outdated (about 20% of the recommendations were out of date within 3 years) [2]. Based on this evidence, 40% (14/35) methodological handbooks typically recommend reviewing and updating guidelines within 2 to 3 years of their publication [6]. Nevertheless, CG developers acknowledge that it is challenging to maintain these predetermined timeframes [7].

### Updating strategy

The updating of CGs is an iterative process that involves an explicit and systematic methodological approach for the identification and assessment of new evidence not included in the original CG [6, 8, 9]. If new relevant evidence is identified and it was considered to have an impact on the current CG, the CG should be modified, if necessary [6, 8, 9]. Moreover, the updating strategies provide an opportunity to improve the overall methodology and edition of the document (e.g. correction of mistakes or enhancement to the writing) [6, 8, 9].

To date, there is no real consensus on the optimal strategy for updating CGs [6, 7, 10]. Most of the available methodological research focuses on identifying new relevant evidence. Research suggests that pragmatic search strategies (with the aim of favouring precision over sensitivity) are efficient and feasible for retrieving new evidence that triggers a recommendation update [6, 11, 12].

### Living clinical guidelines

To address the updating of CGs, the majority of CG developers support the concept of living CGs [7], generally defined as “prospective and active processes that use continuous surveillance and a rapid response to include new relevant evidence identified” [9]. However, until now, no guidance has been developed to put this concept in practice [6], and a few empirical experiences were published [13–17]. CG developers considering the transition to living CGs will have to address challenges to operationalize the process [18].

### Objectives

In order to address some of the challenges related with living CGs, this study aimed to (1) develop a continuous surveillance and updating strategy for CGs and (2) test the strategy in a specific CG.

## Methods

We conducted a cohort study of recommendations from the Assistance During Pregnancy and Puerperium CG included in the Spanish National Health System CG Programme [19].

### Living strategy

The strategy was developed based on published methodological research and the experience of the CG Updating Working Group technical team [2, 6–8, 10, 11, 20, 21]. The processes included in the strategy were as follows: (1) recruitment of members for the CG Updating Working Group, (2) mapping of the CG, (3) identification of evidence from the CG Updating Working Group, (4) designing of restricted literature search strategy, (5) running of restricted literature searches, (6) reviewing alerts for drugs and medical devices, (7) development of reference database, (8) first reference screening, (9) second reference screening (assessment of new evidence impact), (10) development of a clinical questions database, (11) classification of clinical questions, (12) review and, if necessary, modification of clinical questions and recommendations, and (13) update of the CG manuscript (Table 1). Following, we provided a detailed description of the most complex processes: (1) identification of new evidence, (2) reference screening, and (3) classification of clinical questions.

**Table 1:** Description of the continuous surveillance and updating strategy

Process	Description	Participants
1. Recruitment of members for the CG Updating Working Group	− Contact the CG Development Group to invite them to participate in the implementation of the strategy. − Replace non-respondents or those who declined with new members.	− Technical team
2. Mapping of the CG	− Identify clinical questions, recommendations, and references in the CG. − Compile original documentation (searches, references, evidence syntheses, and GRADE evidence profiles).	− Technical team
3. Identification of evidence from the CG Updating Working Group	− Distribute a questionnaire via email among the CG Updating Working Group for identifying new evidence.	− Clinical team − Patients and carers team
4. Design of restricted literature search strategy	− Design and validate restricted search strategies per clinical question. − Design search strategy for costs and resources use and for patients’ values and preferences.	− Technical team
5. Running of restricted literature searches	− Conduct restricted searches in MEDLINE (through PubMed).	− Technical team
6. Review of alerts for drugs and medical devices	− Identify alerts for drugs and medical devices issued by the Spanish Agency for Medicines and Health Products.	− Technical team
7. Development of references database	− Develop references database and identify duplicates among the different information sources and between the original and updated CGs.	− Technical team
8. First reference screening	− Identify *pertinent references* (topic-related references with a fitting study design).	− Technical team
9. Second reference screening (assessment of new evidence impact)	− Develop a questionnaire to identify: (*1*) *relevant references*: references that are pertinent for updating a recommendation but that actually do not trigger an update and (*2*) *potential key references*: references that could potentially trigger an update of a recommendation.	− Clinical team − Technical team
10. Development of a clinical questions database	− Select clinical questions with pertinent, relevant, and key references.	− Technical team
11. Classification of clinical questions	− Analyse clinical questions database to identify: (*1*) *clinical questions to be reviewed*: with potential key references and with different relevant references or important pharmacological alerts, (*2) valid clinical questions*: without potential key references associated and (*3*) *new clinical questions*.	− Technical team
12. Review and, if necessary, modification of clinical questions and recommendations	− Assessment of the potential key references. − Update recommendations if necessary. − Identify key references (references that have triggered changes in one or more recommendations). − Reach a consensus with the CG Updating Working Group on the suggested updates.	− Clinical team − Patients and carers team − Technical team
13. Update of the CG manuscript	− Incorporate updates in the previous version of the CG manuscript.	− Technical team

*CG* clinical guideline

#### Identification of new evidence

Three different strategies were used to identify new evidence: (1) a questionnaire sent to the CG Updating Working Group, (2) a restricted literature search strategy, and (3) a revision of available drugs and medical devices alerts.

The questionnaire sent to the CG Updating Working Group aimed to identify any new relevant evidence that could have an impact on the CG (questionnaire available from the authors upon request). The questionnaire covered the different areas of the CG including the scope, new potential aspects not included in the original version, or new relevant evidence assessing the effectiveness and safety of the interventions. The survey also included questions about other relevant factors such as changes in the relative importance of the outcomes, changes in the resource use and cost of the interventions, equity, acceptability, or feasibility issues that might have arisen since the publication of the CG. Information about on-going research studies was also sought in the survey.

Restrictive literature search strategies were developed for each clinical question in MEDLINE (PubMed) following a validated methodology described elsewhere [11]. In summary, the minimum number of Medical Subject Headings (MeSH) terms and text words required from the original exhaustive search strategies were selected. The strategies were validated checking that all key references supporting the recommendations in the original CG were retrieved and were refined if needed. Once the strategies were validated, PubMed Clinical Queries filters were applied (www.ncbi.nlm.nih.gov/pubmed/clinical). Search strategies by topic were also developed using a specific filter to identify studies on how patients and other stakeholders value health outcomes and economic studies [22, 23].

Finally, drugs and medical devices alerts published by the Spanish Agency for Medicines and Health Products were reviewed (www.aemps.gob.es/en/home.htm).

#### Reference screening

The references were sequentially classified in the following:

- Pertinent references: topic-related references that met the study design criteria
- Relevant references: pertinent references that could be used when considering an update to a recommendation, but that would not necessarily trigger a potential update
- Potential key references: relevant references that could potentially trigger an update

A specific questionnaire for each clinical question was used to identify relevant and key references (questionnaire available from the authors upon request). The questionnaire included the clinical question, the recommendations, and the references considered pertinent in the first screening to that clinical question. If the reference was considered relevant for that particular question by the reviewer, then it was deemed necessary to assess if the reference could potentially trigger an update (key reference). If it was the case, it was necessary to explicitly state which part of the question and/or recommendations was affected (population, intervention, comparator, outcomes, resource use and costs, equity, acceptability, feasibility, strength or direction of the recommendation).

#### Classification of clinical questions

Each clinical question was classified in one of the following categories:

- Clinical question to be reviewed: question with potential key references or with alerts
- Valid clinical question: question without potential key references or without alerts
- New clinical question

Once the questions were classified, we planned to update them following a similar method used in the development of the original recommendations but taking into account the new evidence identified and the evidence used to develop the recommendations.

#### Update cycle

Conducting the 13 processes was considered a one update cycle. The first update cycle included new evidence since the last search date in the CG development process up to the first search date in the CG surveillance process; subsequent update cycles were scheduled every 6 months [2].

### Data analysis

We performed a descriptive analysis of the data: literature search time periods, number of identified references, number of screened references, and number of classified references (pertinent, relevant, and key). We described narratively the steps achieved.

## Results

### Clinical Guideline Updating Working Group

All members of the Pregnancy CG Development Working Group were initially contacted (20 members). However, since only five agreed to participate in this study, 30 additional candidates were contacted. The Pregnancy CG Updating Working Group finally consisted of 29 members: (1) clinical team: three medical specialists in gynaecology and obstetrics, three medical specialists in family and community medicine, and three midwives; (2) patients and caregivers team: three patients or patient representatives; and (3) technical team: 17 CG methodologists.

### Mapping process

We identified 89 clinical questions and 201 recommendations in the Assistance During Pregnancy and Puerperium CG [19]. We focused specifically on the “Assistance during pregnancy” section, which contained 69 clinical questions and 123 recommendations (36 strong recommendations, 49 weak recommendations, and 38 good clinical practice statements). We also retrieved the references used to support recommendations, original literature search strategies, evidence syntheses, and GRADE evidence profiles.

### Continuous surveillance process

We contacted a total of 13 members of the Pregnancy CG Updating Working Group for the baseline survey and received 11 responses (84.6% response rate). We developed one search strategy per clinical question (a total of 62, as 7 clinical questions were clustered together), as well as topic searches for studies on patients’ values and preferences and for costs and resource use.

We identified 26 recommendations (26/123; 21.1%) related to drugs or dietary supplements. We consulted drugs and medical devices alerts from the Spanish Agency for Medicines and Health Products, searching by CG as topic.

For the first literature search cycle (32-month period), 9710 references were identified. Of these, the technical team classified 318 as pertinent, 289 as relevant, and 55 as potential key references (Table 2).

**Table 2:** Preliminary results of the continuous surveillance implementation

	First update cycle	Second update cycle	Third update cycle
Literature search			
–Search dates	01/01/2012 31/08/2014	01/09/2014 28/02/2015	01/03/2015 31/08/2015
–Time period included (months)	32	6	6
Results of the literature search			
–Evidence identified from the CG Updating Working Group	19	NC	NC
–References on efficacy	9191	2089	1946
–References on costs and resource use	116	51	19
–References on patients’ values and preferences	384	10	39
–Drug alerts	NA	10	6
Total	9710	2160	2010
Results of reference screening			
–Pertinent references	318	NC	NC
–Relevant references	289	NC	NC
–Potential key references (≥1 participants)	184	NC	NC
–Potential key references (≥2 participants)	31	NC	NC
–Potential key references (CG methodology experts)	55	NC	NC

*NA* not available, *NC* not completed

For the second and third literature search cycles (each a 6-month period), 2160 and 2010 references were retrieved, respectively (Table 2).

The surveillance process lasted 1 year, from November 2014, when the CG development institution was contacted for establishing the Pregnancy CG Updating Working Group, until November 2015, when the study was stopped early due to budgetary constraints.

The continuous surveillance and updating strategy has not yet been completely implemented. We have not assessed the results of the second and third cycles of the literature search or gauged the effect on recommendations of potential key references. As such, we have not reviewed and, if necessary, updated the CG recommendations.

## Discussion

### Main findings

We designed a step-by-step process for continuous surveillance and updating CGs. We were able to implement a continuous and restricted literature search strategy for the “Clinical Practice Guideline on Assistance during Pregnancy and Puerperium” for a 1-year period. In the first update cycle we identified 9710 references (318 pertinent, 289 relevant, and 55 potential key references). For the second and third update cycles 2160 and 2010 references were retrieved, respectively.

The continuous surveillance and updating strategy has not yet been completely implemented due to budgetary constraints.

### Our results in the context of previous research

Only one previous study, published in 2003, assessed a continuous surveillance and updating strategy for CGs, specifically for cancer guidelines. This approach included a continuous and exhaustive literature search strategy, evaluation of the newly found evidence, review and updating of recommendations, and dissemination of the new evidence and modified recommendations among stakeholders. Similarly to our experience, the authors of this study highlighted the considerable resources required [17].

Other initiatives have ventured the implementation of new technologies to facilitate the CG updating process. One of them, called MAGIC (Making GRADE the Irresistible Choice) provides a publication platform where the main content of CGs can be disseminated. MAGIC also facilitates uploading modifications, including any potential updates, which would be available to users instantly [24]. Similarly, “Kidney Disease: Improving Global Outcomes (KDIGO)” recently published a series of recommendations for a continuous, dynamic strategy for maintaining their CGs current. Their model heavily relies on the availability and processing of new evidence using integrated electronic platforms [25]. Unfortunately, these new technologies have not yet been formally implemented and evaluated.

### Strengths and limitations

We were able to retrieve, organise, and map the original documentation related to the development of the assessed CG, including the clinical questions, recommendations and references, original literature search strategies, evidence syntheses, and GRADE evidence profiles. We also adopted a systematic and continuous approach (every 6 months) to identify new evidence and to assess its impact on the CG recommendations. Lastly, we introduced evidence searches for patients’ values and preferences and for costs and resource use in the surveillance process.

However, our work is subject to some limitations. First of all, we have not been able to assess the impact of the new evidence on clinical questions and recommendations for either the second or third update cycles. In addition, we have not reviewed or modified clinical questions and recommendations based on the identified new evidence in any of the update cycles.

Second, we had difficulties assembling the Pregnancy CG Updating Working Group. The vast majority of the CG Developing Group did not take part in the implementation of the strategy. Hence, an almost new working group had to be set up for this purpose. On the other hand, some members of the Pregnancy CG Updating Working Group withdrew during the study, probably due to an excessive study-related workload (appraisal of a high volume of publications in the first update cycle, inadequate training related to the implementation of the strategy, and/or a lack of knowledge of the content of the original CG).

Third, the first surveillance cycle was quite resource-intensive (from the last search date in the original CG development process to the first search date in the CG surveillance process) and required the retrieval, mapping, and classification of the documentation generated during the development of the original CG. The process was optimised in the subsequent update cycles (second and third) given that (1) the process had already started and (2) the time between cycles (6 months) and, consequently, the volume of references (approx. 2000 references) were smaller.

Lastly, and related to the previous limitation, we did not have adequate funding to take on the management and development of the completely continuous surveillance and updating strategy we originally intended to implement. CG developers should consider using different surveillance and updating strategies to maintain their CGs up-to-date (a living strategy might not be suitable for all CGs). More research is needed to identify which CGs, topics, or areas could benefit from this or other approaches.

## Conclusions

Implementing a continuous and restricted literature search process is a potentially feasible approach for the surveillance of new evidence. A continuous surveillance and updating strategy (as living CG) requires long-term substantial resources for its adoption. Further resources are needed in the updating CG field, both for implementing updating strategies and for developing methodological research.

## Abbreviations

CG: Clinical guideline
